# A first case report of dapsone inducing recurrent ventricular arrhythmia

**DOI:** 10.1093/ehjcr/ytz158

**Published:** 2019-09-20

**Authors:** Bethany Wong, Lavanya Saiva, John Buckley, Joseph Galvin

**Affiliations:** Cardiology Department, Connolly Hospital, Mill Rd, Abbotstown, Dublin 15, 15 X40D, Ireland

**Keywords:** Case report, Dapsone, Ventricular arrhythmia, Cardiomyopathy, Drug

## Abstract

**Background:**

Ventricular arrhythmias (VAs) are life-threatening arrhythmias which are associated with significant morbidity and mortality. Ventricular arrhythmias are induced by a change in the myocardial environment altering cardiomyocyte electrophysiology. The substrate for VA includes myocardial scar, electrolyte disturbances, and drugs altering cellular electrophysiology.

**Case summary:**

Here, we present a case of a 52-year-old man with known ischaemic cardiomyopathy, presenting with VA storms secondary to dapsone, an anti-microbial used in this case for the prophylaxis of pneumocystis pneumonia. This is the first case linking dapsone to the development of VAs. Ventricular arrhythmias storm occurred towards the end of the course of anti-microbial therapy and the patient was referred for sympathectomy. However, following the end of treatment, no further VA occurred and sympathectomy was therefore avoided.

**Discussion:**

The underlying mechanism for the association between dapsone treatment and VA is unclear and a prolonged QTc was not observed in our case. It is important to recognize that every drug has many physiological effects and in patients with underlying diseases whereby there is already an unfavourable environment, additional drugs can lower the threshold of triggering an arrhythmia and the result can be life-threatening. In a patient with ischaemic cardiomyopathy, where underlying substrate for VA may already exist, the introduction of dapsone could lower the threshold for development of arrhythmia.

**Learning points**

- Dapsone has the potential to cause ventricular arrhythmias in patients with ischaemic cardiomyopathy.
- When a patient’s care is at the end stages of recognized treatment to consider withdrawal of medication as an appropriate action.

## Introduction

There are over 4 million global deaths per year due to sudden cardiac death and ventricular arrhythmia (VA).^1^ The substrates for VA include scar-related re-entry, prolonged QT_C_ and drugs.^1^^,^^2^ Here, we present a first case presentation of dapsone reducing the threshold of VA, leading to intractable, recurrent VA despite maximal pharmacological anti-arrhythmic agents. The underlying mechanism by which dapsone acted as a substrate for VA is not known but prolongation of the QT interval was not seen in this case.

## Timeline

**Table:** 

Day	Events
0	A 52-year-old man was admitted with pneumocystis pneumonia, requiring inotropic, and ventilatory support. He was discharged on dapsone 100 mg prophylaxis. During admission, interrogation of his implantable cardioverter-defibrillator (ICD) showed multiple episodes of non-sustained ventricular tachycardia; non-requiring ICD therapy.
14	One appropriate ICD shock for ventricular fibrillation. Oral anti-arrhythmics uptitrated.
35–56	Recurrent polymorphic ventricular tachycardia requiring six shocks from his ICD despite optimized oral and intravenous anti-arrhythmics and revascularization. The patient was accepted for end-stage treatment with cervical sympathectomy. Whilst waiting for the surgery, medications were reviewed and dapsone alone was held. After 96 h, he had no further ventricular arrhythmia (VA). Cervical sympathectomy was avoided and he was discharged.
6 months later	No further episodes of VA detected.

## Case presentation

**Figure 1:**
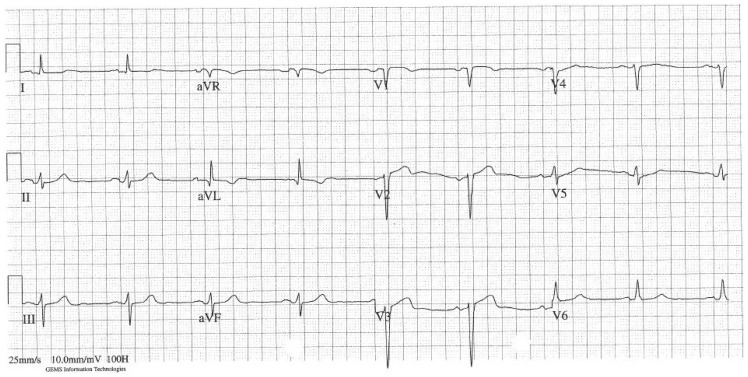
The resting 12-lead electrocardiogram showing sinus bradycardia and a normal QTc.

A 52-year-old taxi driver with a background history of ischaemic cardiomyopathy with a primary prevention implantable cardioverter-defibrillator (ICD) and pancreatic cancer under surveillance after chemotherapy, presented 2 weeks after initiation of dapsone with ventricular fibrillation (VF) requiring an ICD shock. At this stage, he had recently been discharged from hospital after being treated for pneumocystis pneumonia, which had required intubation and prophylaxis of subsequent infections with dapsone. Initial examination and investigations were normal with no evidence of electrolyte disturbance or infection. Transthoracic echocardiogram on presentation showed an unchanged ejection fraction of 30%. *Figure 1* shows his resting electrocardiogram. The ICD shock was treated with uptitration of his mexiletine from 100 to 200 mg b.i.d. and he was subsequently discharged. He represented 5 weeks later with further VF requiring an ICD shock. Initial investigations showed no specific substrate and his mexiletine dose was increased to 300 mg t.d.s. He was discharged and represented a third time with further VF episodes and he was initiated on an amiodarone infusion.

**Figure 2:**
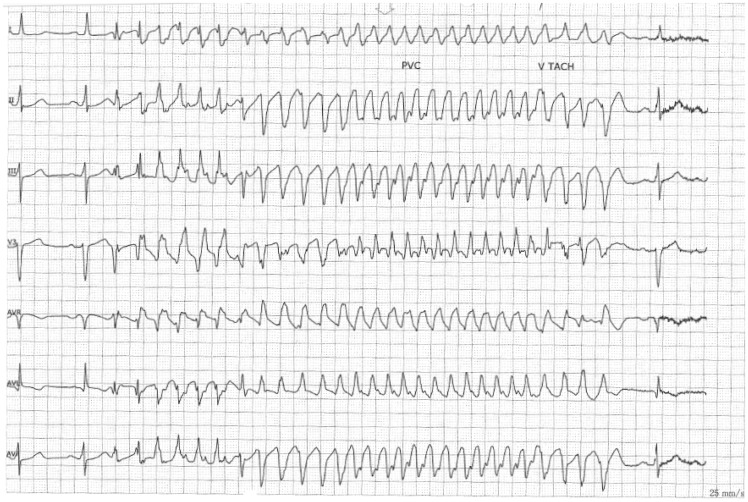
A rhythm strip showing the onset of ventricular fibrillation.

**Figure 3:**
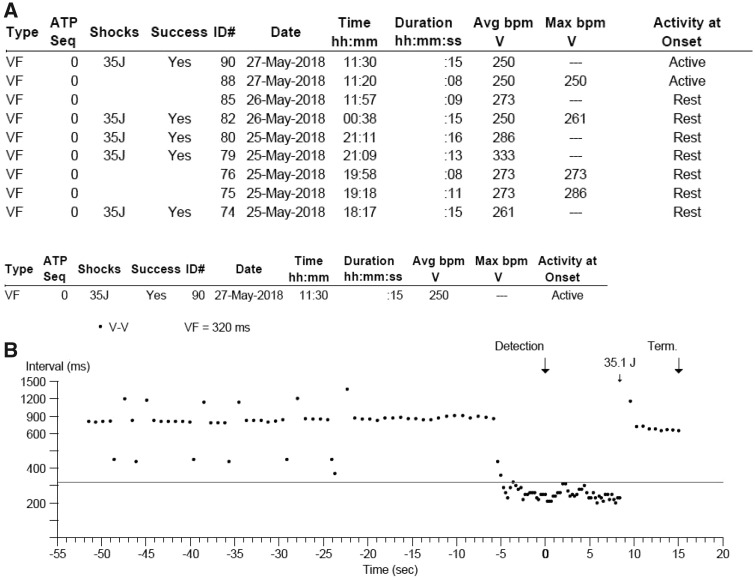

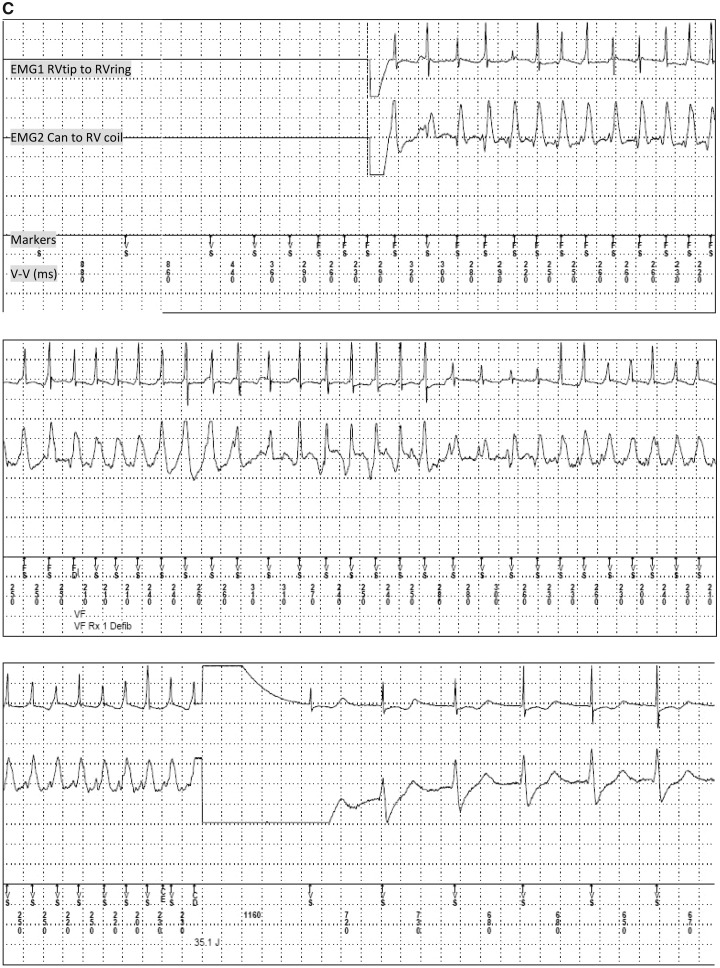
An example of an implantable cardioverter-defibrillator interrogation showing multiple ventricular arrhythmias requiring shocks. (*A*) Overview of an implantable cardioverter-defibrillator interrogation over a 48 h period where nine episodes of ventricular arrhythmias were detected. Five were terminated with shocks after they reached 15 s in duration. (*B*) Ventricular arrhythmias detection demonstrated on an R-R interval graph, where the threshold of 320 ms is reached and ventricular arrhythmias detected. It was subsequently terminated with a 35.1 J shock. (*C*) Electromyography of ventricular arrhythmias detection and treatment with a shock. EMG, electromyography; FS, fibrillation sense; J, joule; RV, right ventricle; VS, ventricular sense.

He continued to have multiple ICD shocks secondary to VF, whilst on the amiodarone infusion and so this was switched to a lidocaine infusion. *Figure 2* shows an example of captured VA. *Figure 3* demonstrates his ICD interrogation. During each admission with VA, it was noted he was also not doing any physical exercise during the shocks and the episodes did not have a temporal concordance with daily medications. The patient also did not have any additional symptoms other than those related to the VA on any of the occasions pre-admission. On the third admission, to exclude an ischaemic cause, he had an angiogram which showed a moderate small mid-right coronary artery with a 70% narrowing, which was stented in the absence of any other reversible causes.

He was discharged but unfortunately was readmitted 3 days later with a collapse and polymorphic ventricular tachycardia (VT). He was recommenced on a lidocaine infusion but because of continued VAs, he was listed for a cervical sympathectomy. Cervical sympathectomy acts by reducing the effect of sympathetic hyperactivity on the myocardium. Studies have demonstrated that there is hyperinnervation at the border of the normal myocardium and scars which acts to increase the action of the sympathetic overdrive seen in VA.^3^ There is now evidence that video-assisted thoracoscopic sympathectomy is a safe and effective treatment to reduce VA burden in patients with life-threatening arrhythmias due to catecholaminergic polymorphic VT or long QT syndrome.^3^ However, in ischaemic cardiomyopathy, there are currently no guidelines to recommend cervical sympathectomy.^1^ Ablation was not possible in this gentleman as the VT was polymorphic. Pacing the atria to achieve less ectopy via the ICD was not considered as he did not have an atrial lead. A cardiac magnetic resonance imaging to assess for scar burden and inducible ischaemia was not possible due to a non-compatible ICD. At this stage, his medications were re-reviewed with an open mind and dapsone was stopped in case there was any interaction that had not been recognized. He had no further runs of VA and was discharged home without the need for the sympathectomy. He was not changed onto a different prophylactic antibiotic agent, as he had a previous severe allergy to the alternative option: co-trimoxazole. During this month of admission for VA, he did not receive any chemotherapy, antibiotics, or other drugs not mentioned that could have contributed to a pro-arrhythmogenic environment. There was also no evidence of decompensated heart failure, electrolyte, or hormone imbalance during this time. His QTc was 420–450 ms throughout the admissions. He has subsequently been VA free for 6 months with a reducing regime of amiodarone.

## Discussion

In the literature, the effect of dapsone on the heart has only been described twice.^4^^,^^5^ Both have been clinical case reports of patients who have developed dapsone hypersensitivity syndrome. This syndrome is an idiosyncratic drug reaction which occurs in 0.5–3.6% of patients treated with dapsone. It is a triad of fever, rash and systemic involvement; usually, hepatic and haematological. In these two patients, dapsone caused myocarditis; in one case, this was fatal. In our case report, there was no evidence of dapsone hypersensitivity syndrome and a different mechanism of cardiac involvement is likely to have been involved whilst inducing VA.

Dapsone, a synthetic derivative of diamino-sulfone has anti-inflammatory and anti-bacterial properties. It is commonly used in the treatment of mycobacterium leprosae, pneumocystitis pneumonia, and dermatological conditions such as dermatitis herpetiformis. It inhibits dihydropteroate synthase, an enzyme important in folate synthesis which subsequently interferes with DNA synthesis. Dapsone also interferes with myeloperoxidase (important in oxidative stress pathways) in neutrophils and inhibits chemotaxis in neutrophils. This is the likely mechanism for its anti-inflammatory effects.^6^

Interestingly, animal studies have shown inhibition of neutrophils and myeloperoxidase is VA protective. Studies using CD11b/CD18-integrin-deficient mice, subjected to ischaemia and reperfusion show reduced neutrophil infiltration and cardiac myeloperoxidase deposition was significantly protective against VA.^7^^,^^8^ This was also the case with knockout myeloperoxidase rabbits which have shown an alteration of cardiomyocyte ion channels, specifically a reduction in action potential amplitude and a reduction in VA.^7^^,^^8^ These studies have however been in Phase 2 arrhythmia post ischaemia (subacute), which may or may not have been important in our case, as he was developing VA’s without any ischaemic event. There have not been any studies researching the effects of neutrophils/myeloperoxidase on VA in non-ischaemic, scar burdened cardiomyocyte models. It is also important to note that the pathways of oxidative stress are complex, not yet fully elucidated and the interaction of dapsone on folate and myeloperoxidase on cardiomyocytes has not yet been explored.

In cardiomyopathy patients, there is often an unfavourable environment and a small change in this can lead to arrhythmias. This case highlights the importance of questioning all prescribed medications for the safety of our patients especially in cases where there are life-threatening intractable arrhythmias.

## Patient perspective

It is well established that ICD shocks can cause significant psychological stress.^9^ The majority of his ICD shocks occurred when he was unconscious, which may have reduced his anxiety and fear. Despite the multiple ICD shocks and re-admissions, our patient states: ‘on reflection, I felt an over-riding theme of reassurance, confidence in my management and a personalized service’. He felt he had an excellent rapport and trust in the consultants and team looking after him. He states that they all knew his case very well and the communication and management were excellent. In his words: ‘Out of their work, I am still alive’.

## Lead author biography

**Figure:**
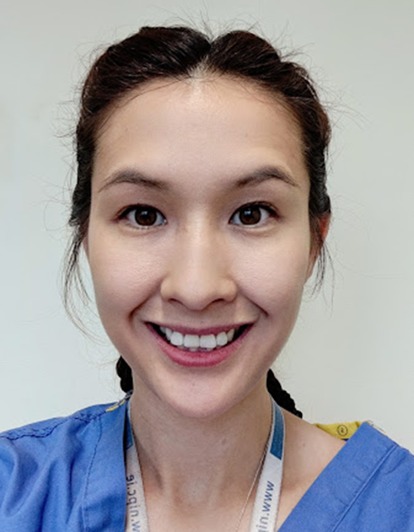


Dr Bethany Wong is a cardiology specialist registrar and lecturer for Royal College of Surgeons in Dublin, Ireland. She graduated from Imperial College School of Medicine and obtained First Class honours in her BSc in cardiovascular sciences. She has authored research in both basic science and clinical cardiology including in transcriptional and translational regulation in cardiomyocytes exposed to oxidative stress and in balloon valvuloplasty in transcatheter aortic valve implantation patients. She is currently completing a diploma in health professions education to extend her interest in teaching.

## Supplementary material

Supplementary material is available at *European Heart Journal - Case Reports* online.

**Slide sets:** A fully edited slide set detailing this case and suitable for local presentation is available online as Supplementary data.

**Consent:** The author/s confirm that written consent for submission and publication of this case report including image(s) and associated text has been obtained from the patient in line with COPE guidance.

**Conflict of interest:** none declared.

## Supplementary Material

ytz158_Supplementary_Slide_Set
