# Supplementary material for: A first case report of dapsone inducing recurrent ventricular arrhythmia
Source: Eur Heart J Case Rep. 2019 Sep 20;3(4):1–6. doi: 10.1093/ehjcr/ytz158 (PMC6939790; doi:10.1093/ehjcr/ytz158)
Supplement: ytz158_Supplementary_Slide_Set [file ytz158_supplementary_slide_set.pptx]

## Slide 1
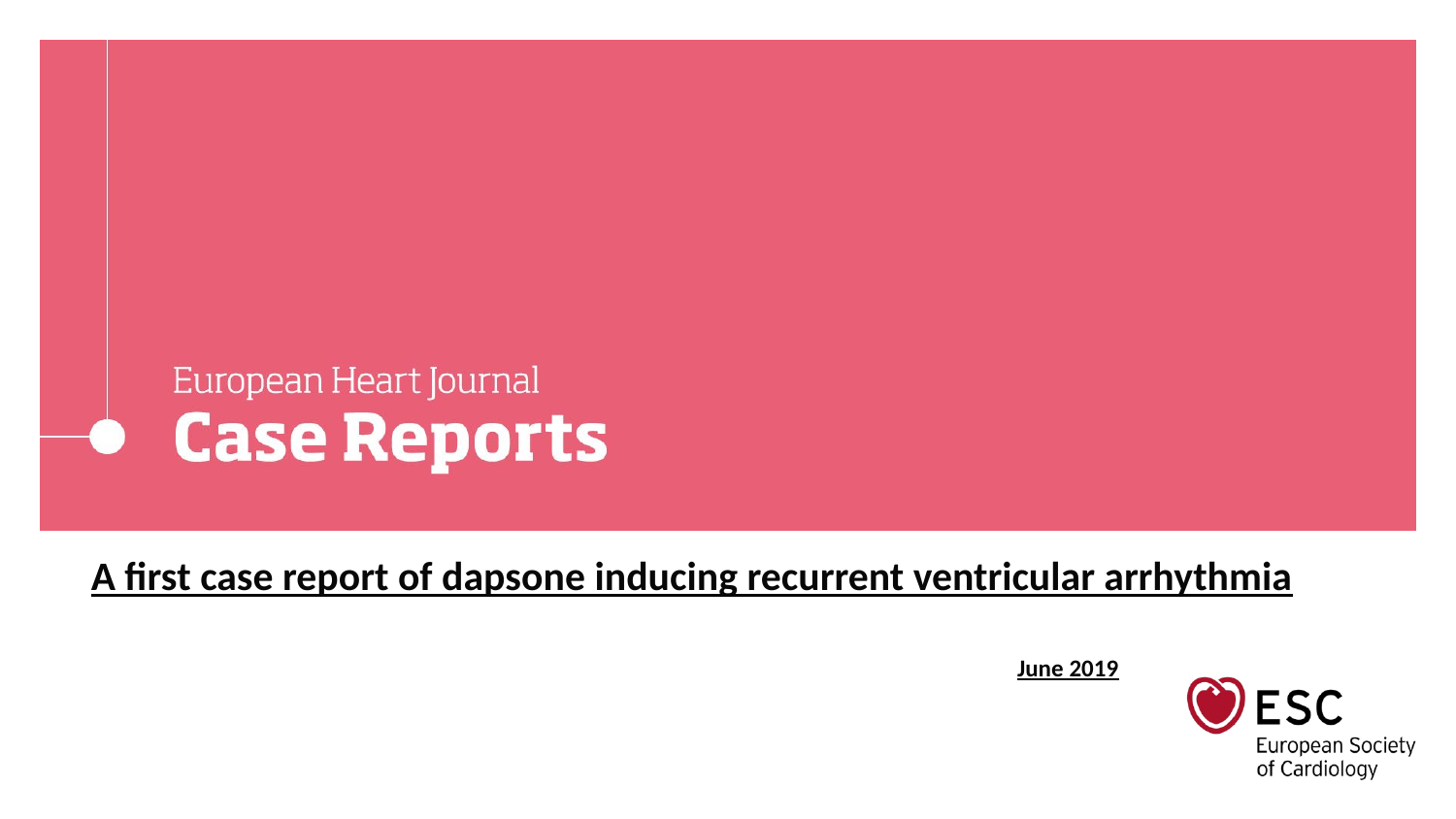

# A first case report of dapsone inducing recurrent ventricular arrhythmia
June 2019

## Slide 2
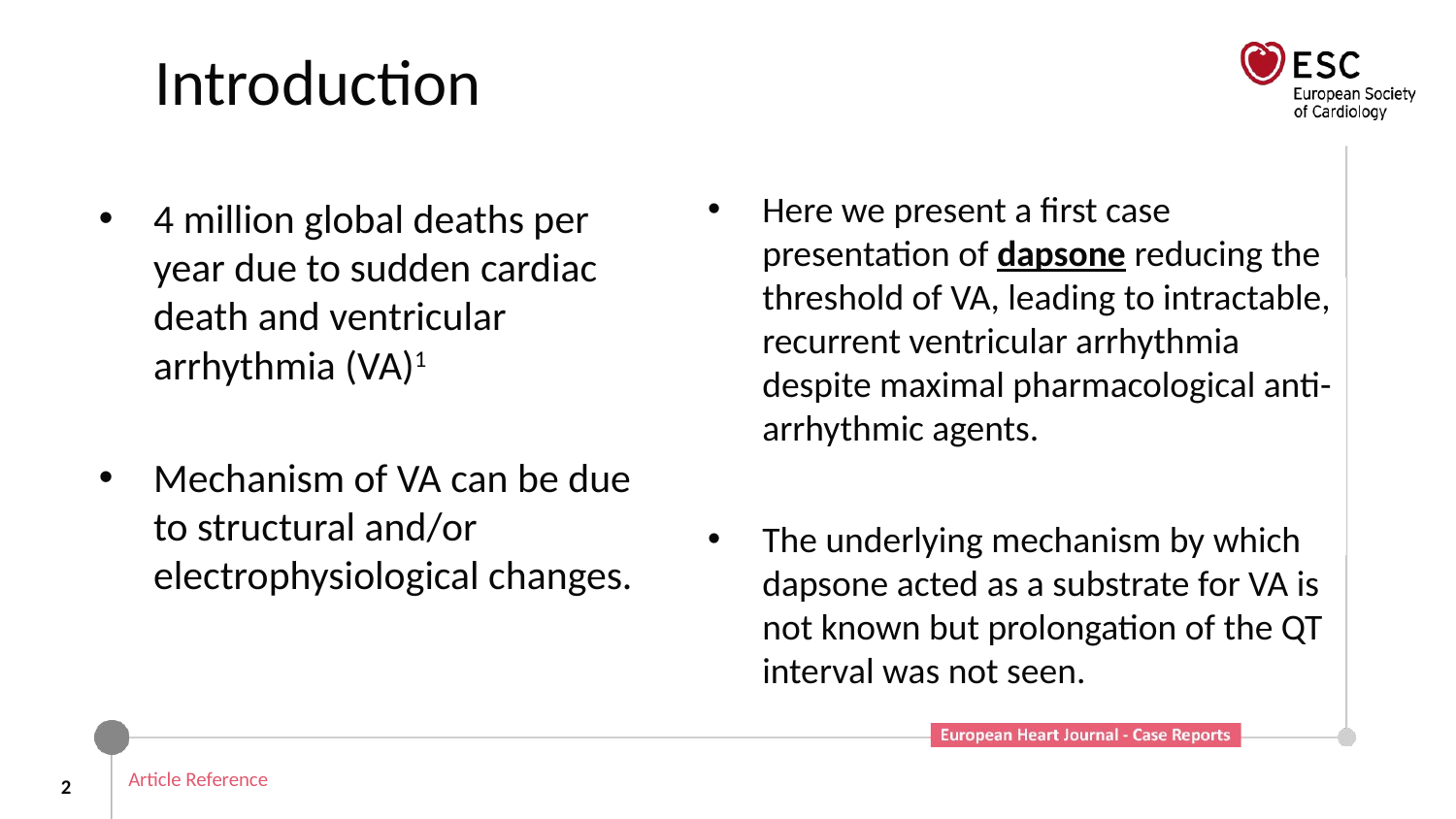

# Introduction
Here we present a first case presentation of dapsone reducing the threshold of VA, leading to intractable, recurrent ventricular arrhythmia despite maximal pharmacological anti-arrhythmic agents.
The underlying mechanism by which dapsone acted as a substrate for VA is not known but prolongation of the QT interval was not seen.
4 million global deaths per year due to sudden cardiac death and ventricular arrhythmia (VA)1
Mechanism of VA can be due to structural and/or electrophysiological changes.
Article Reference
2

## Slide 3
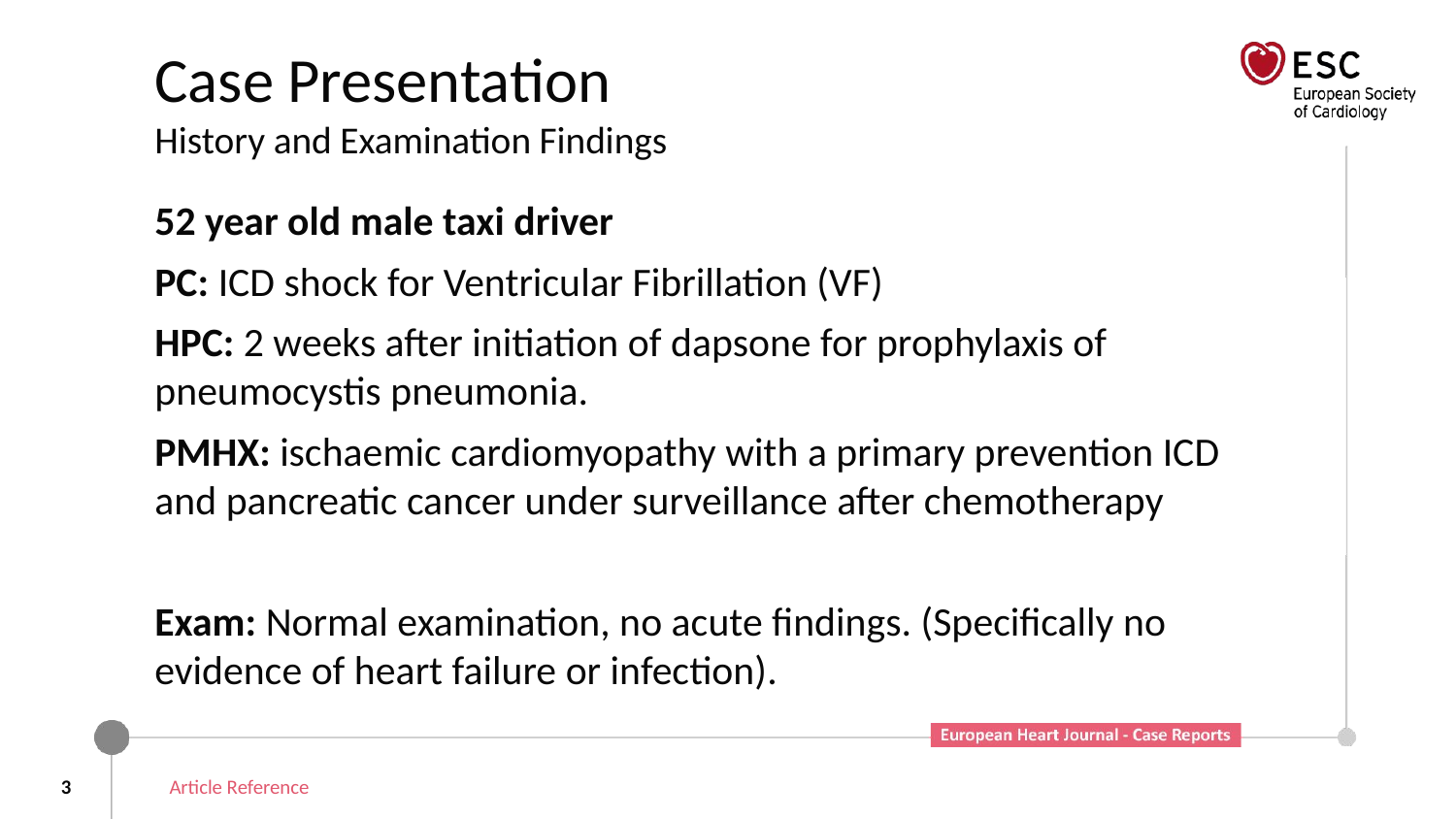

# Case Presentation History and Examination Findings
52 year old male taxi driver
PC: ICD shock for Ventricular Fibrillation (VF)
HPC: 2 weeks after initiation of dapsone for prophylaxis of pneumocystis pneumonia.
PMHX: ischaemic cardiomyopathy with a primary prevention ICD and pancreatic cancer under surveillance after chemotherapy
Exam: Normal examination, no acute findings. (Specifically no evidence of heart failure or infection).
3
Article Reference

## Slide 4
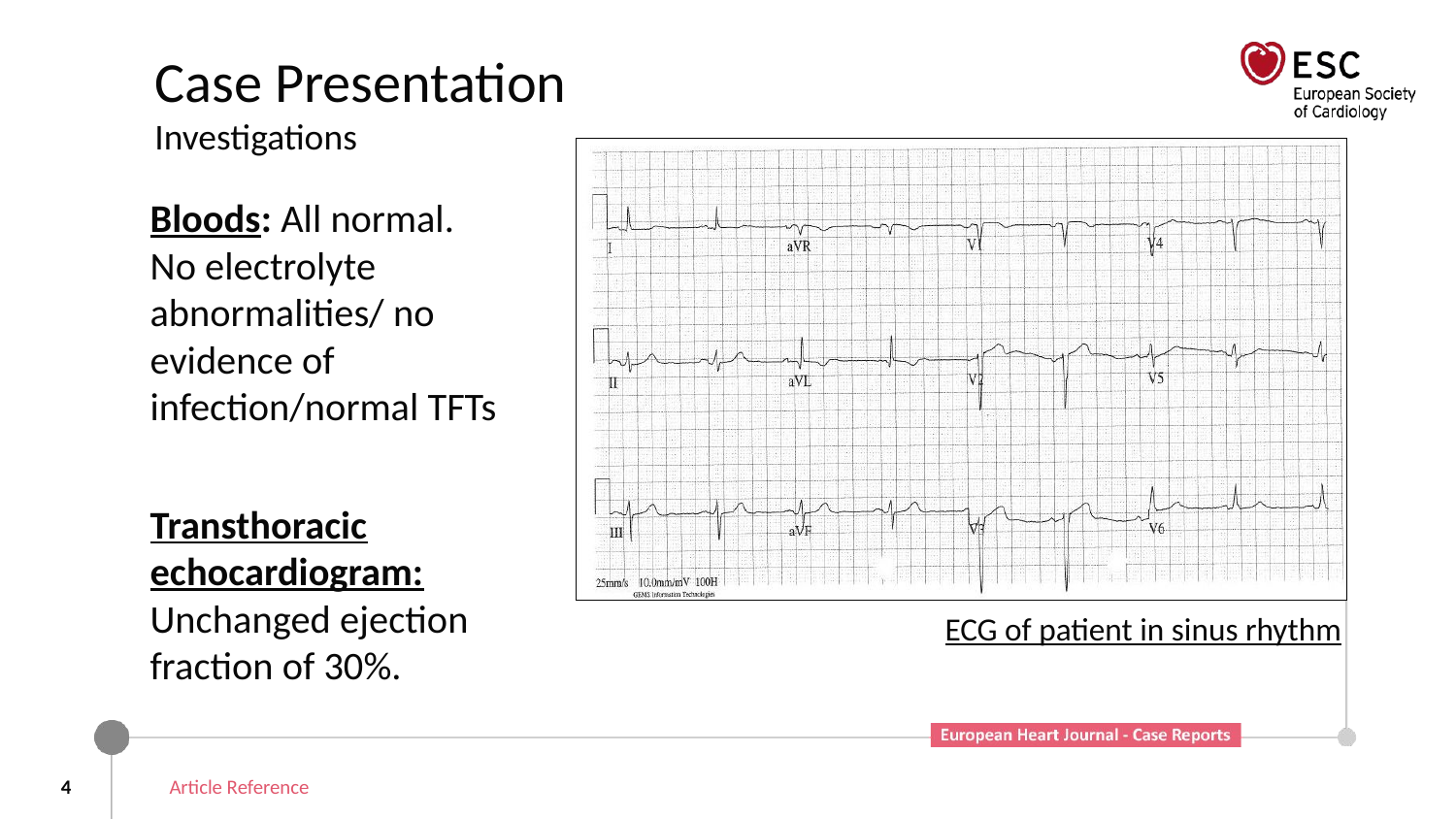

# Case Presentation Investigations
Bloods: All normal. No electrolyte abnormalities/ no evidence of infection/normal TFTs
Transthoracic echocardiogram: Unchanged ejection fraction of 30%.
ECG of patient in sinus rhythm
4
Article Reference

## Slide 5
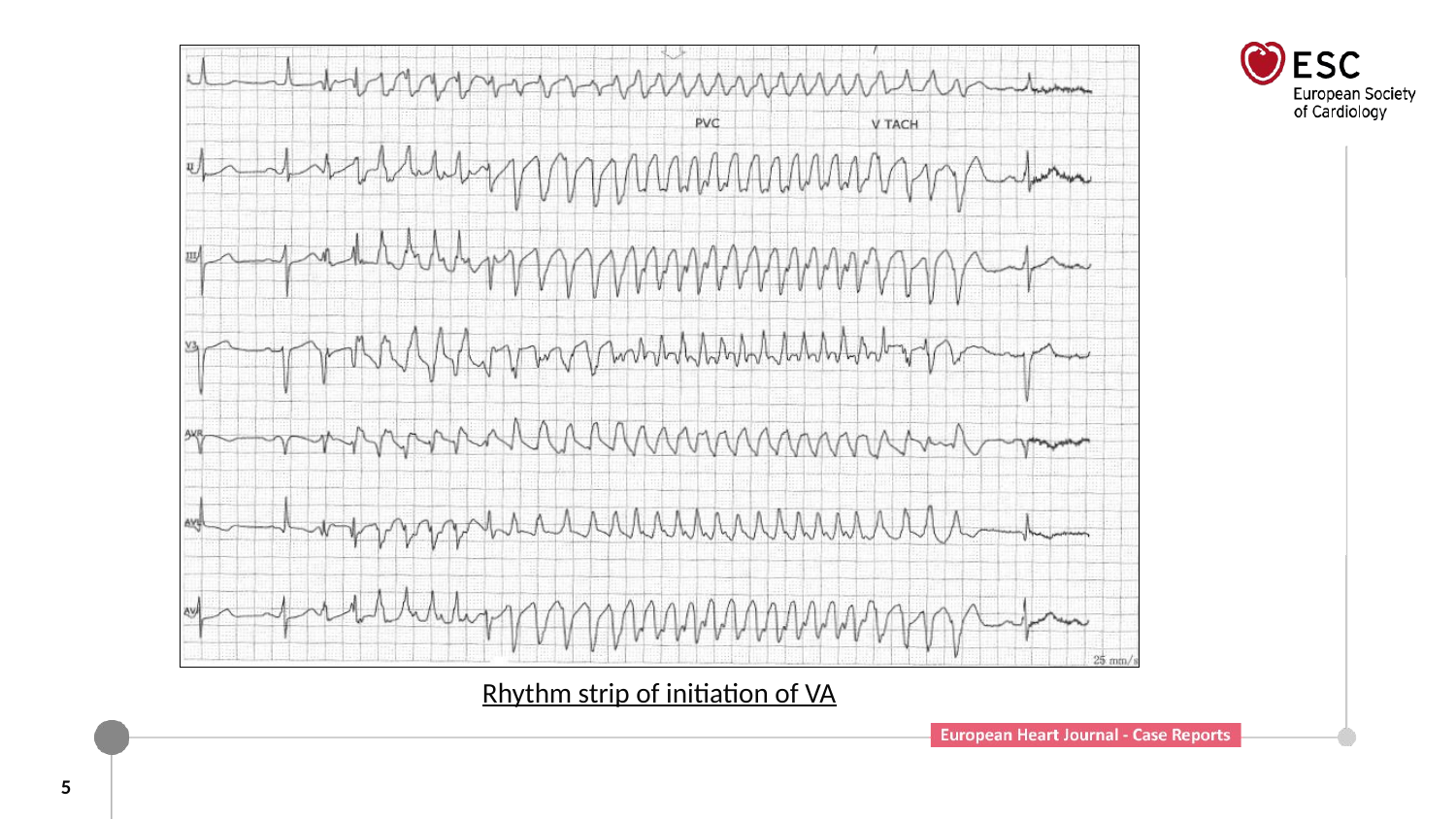

Rhythm strip of initiation of VA
5

## Slide 6
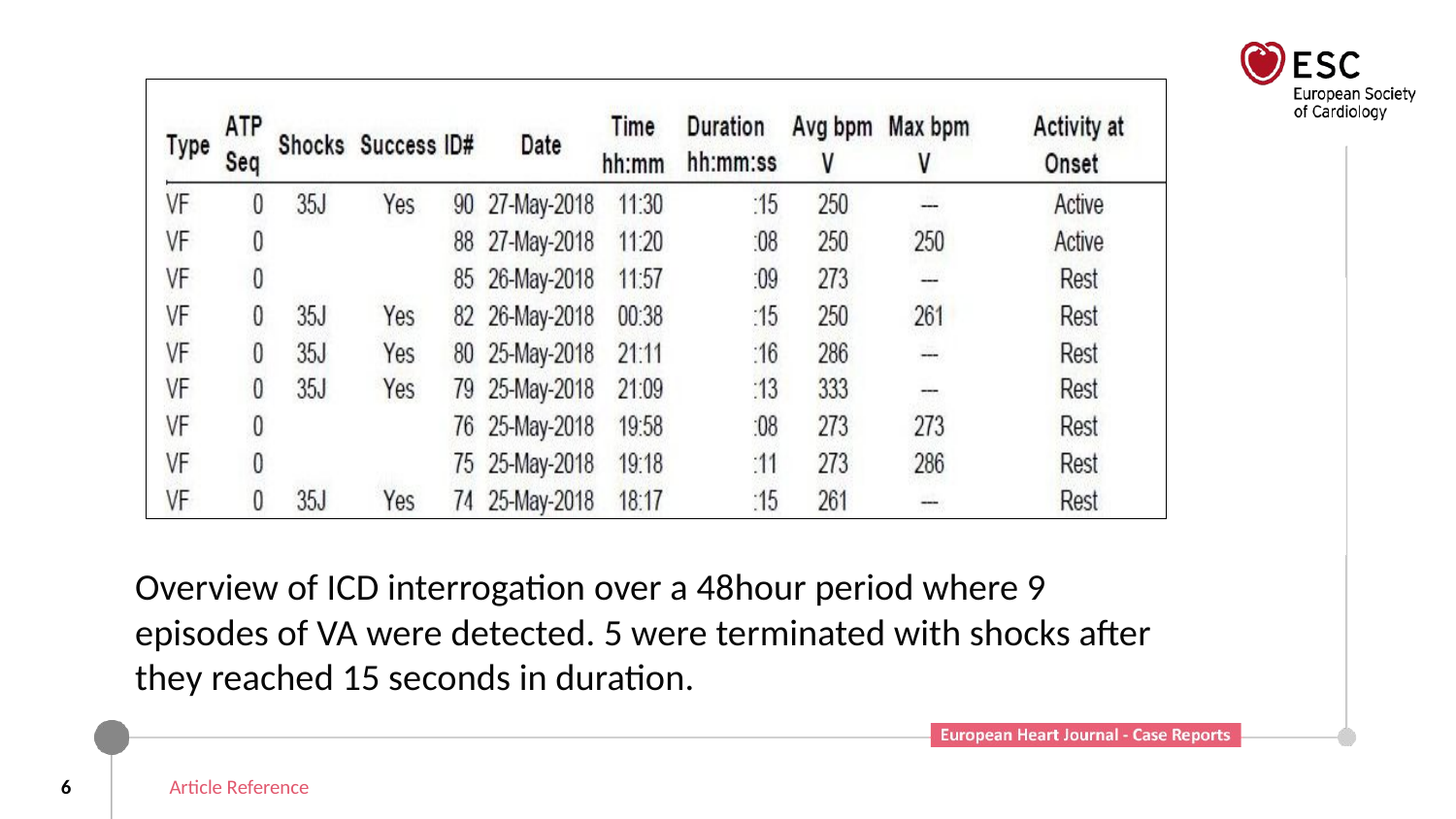

Overview of ICD interrogation over a 48hour period where 9 episodes of VA were detected. 5 were terminated with shocks after they reached 15 seconds in duration.
6
Article Reference

## Slide 7
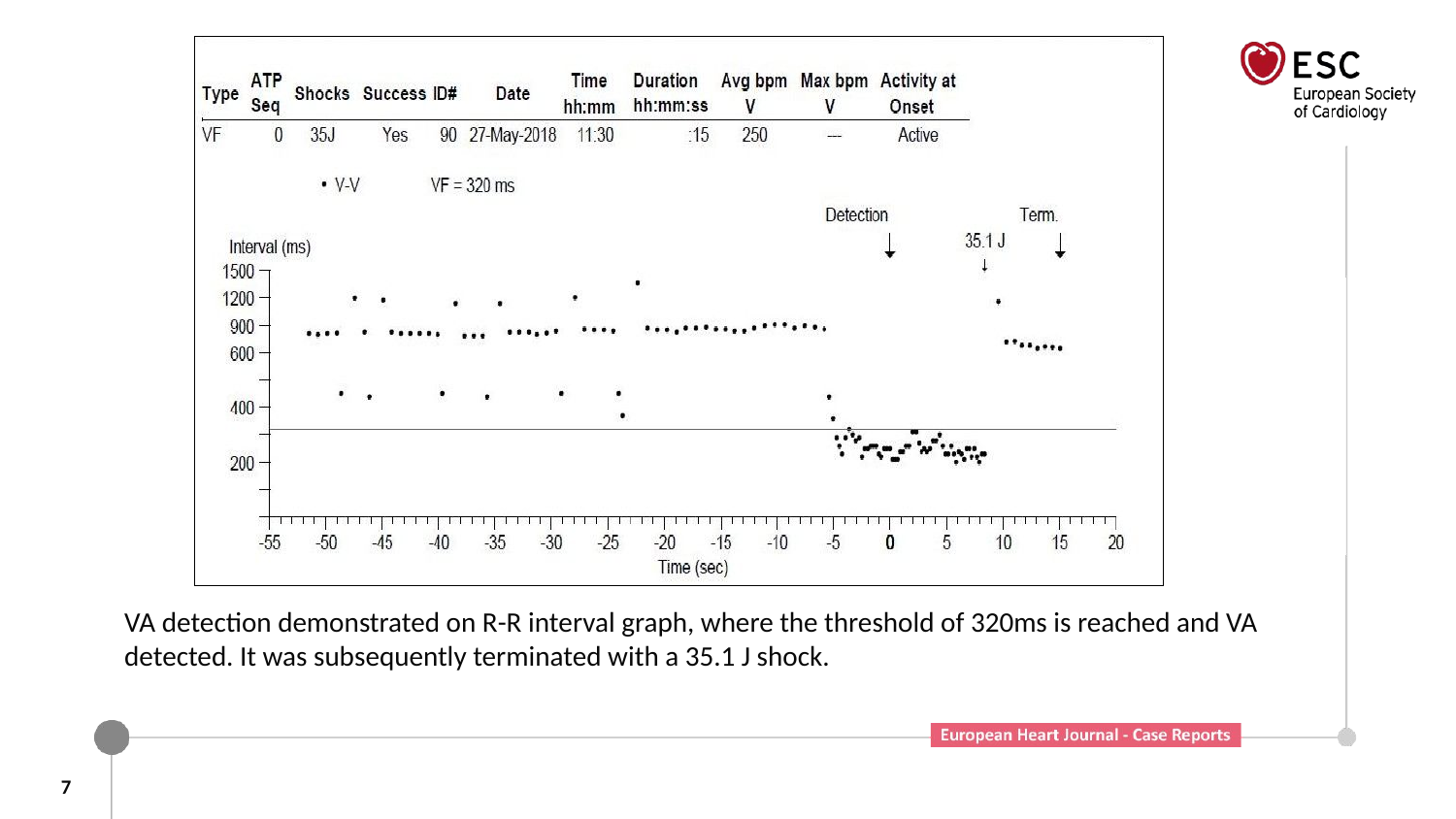

VA detection demonstrated on R-R interval graph, where the threshold of 320ms is reached and VA detected. It was subsequently terminated with a 35.1 J shock.
7

## Slide 8
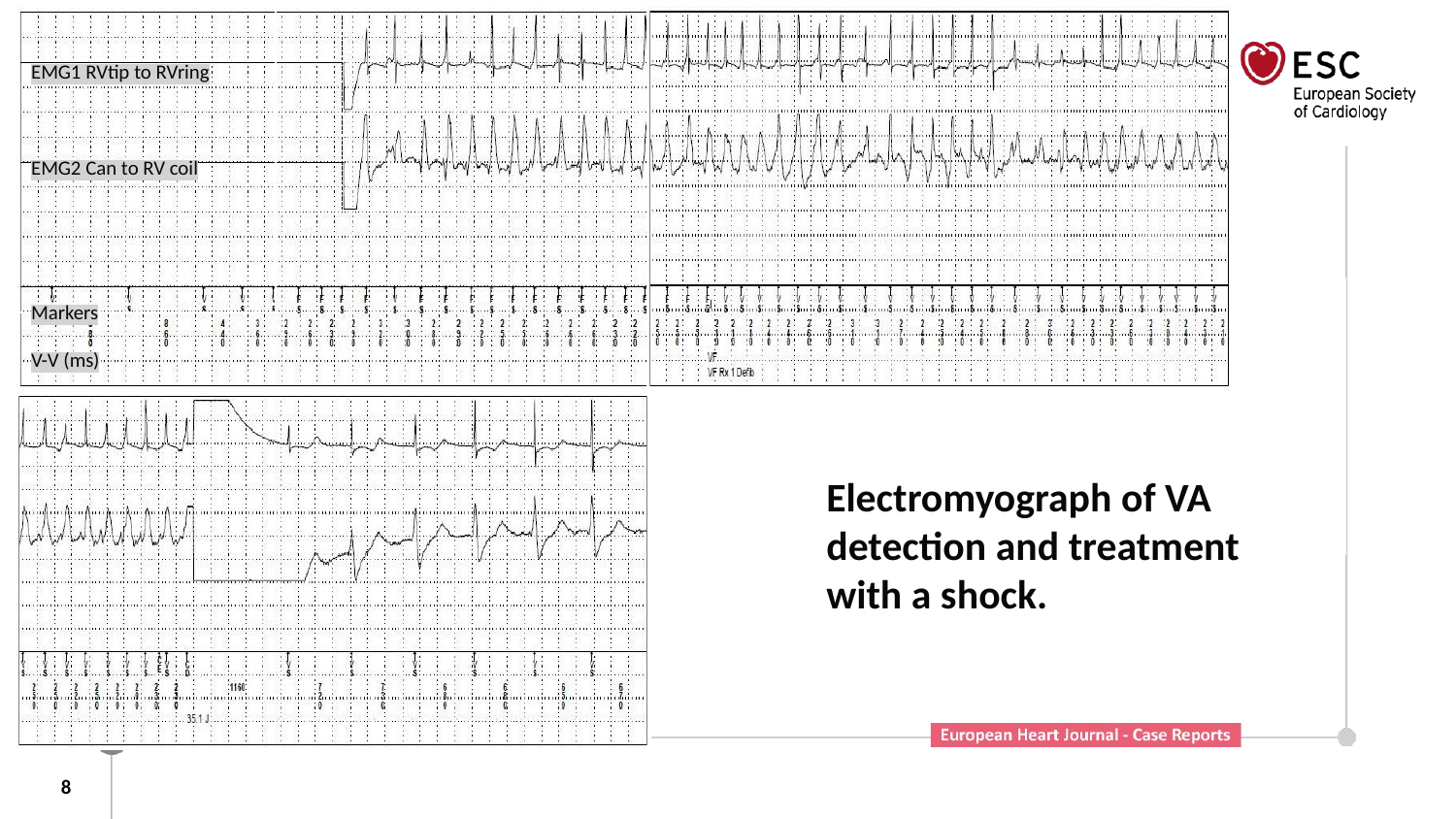

EMG1 RVtip to RVring
EMG2 Can to RV coil
Markers
V-V (ms)
Electromyograph of VA detection and treatment with a shock.
8

## Slide 9
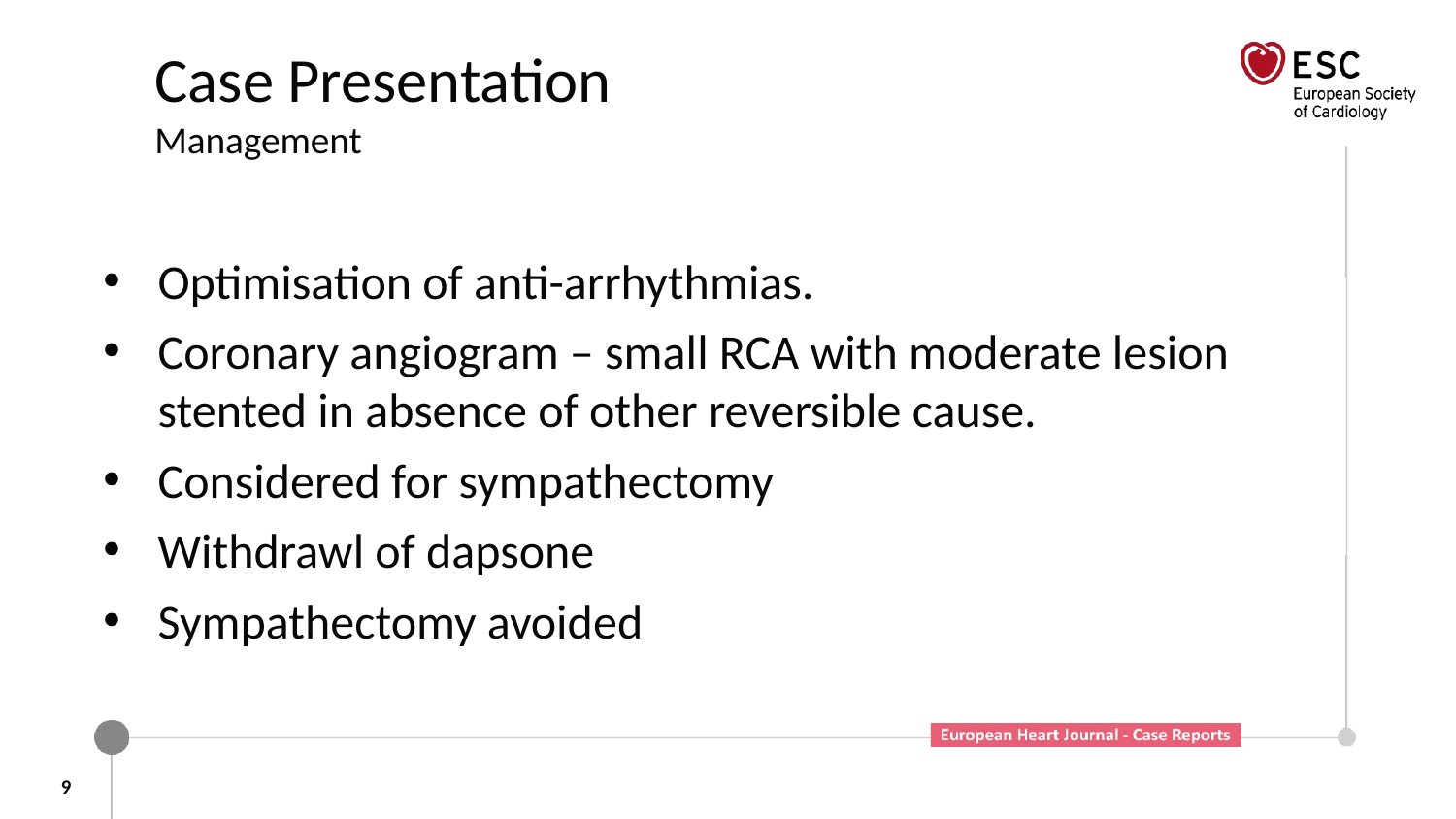

# Case Presentation Management
Optimisation of anti-arrhythmias.
Coronary angiogram – small RCA with moderate lesion stented in absence of other reversible cause.
Considered for sympathectomy
Withdrawl of dapsone
Sympathectomy avoided
9

## Slide 10
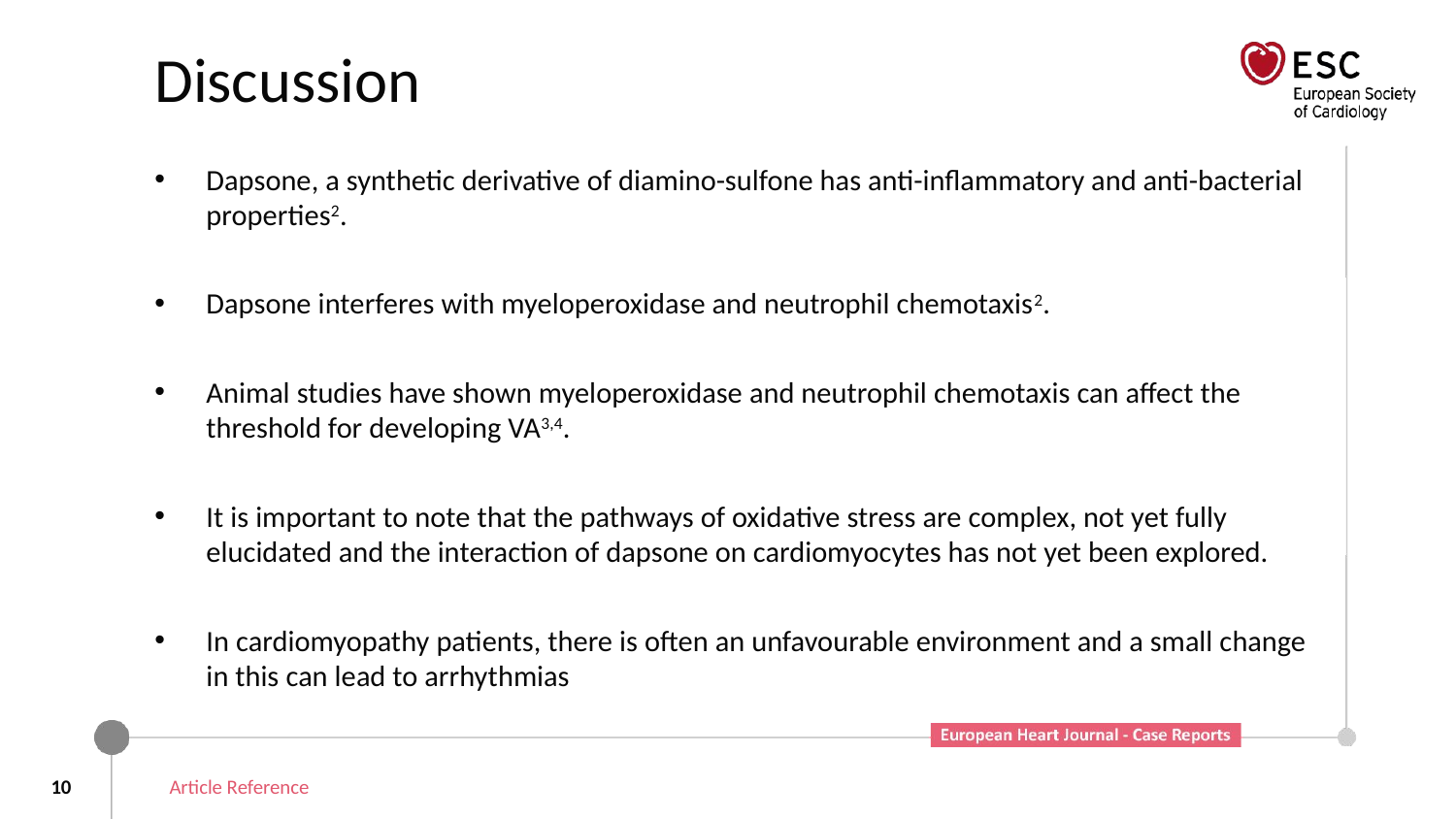

# Discussion
Dapsone, a synthetic derivative of diamino-sulfone has anti-inflammatory and anti-bacterial properties2.
Dapsone interferes with myeloperoxidase and neutrophil chemotaxis2.
Animal studies have shown myeloperoxidase and neutrophil chemotaxis can affect the threshold for developing VA3,4.
It is important to note that the pathways of oxidative stress are complex, not yet fully elucidated and the interaction of dapsone on cardiomyocytes has not yet been explored.
In cardiomyopathy patients, there is often an unfavourable environment and a small change in this can lead to arrhythmias
10
Article Reference

## Slide 11
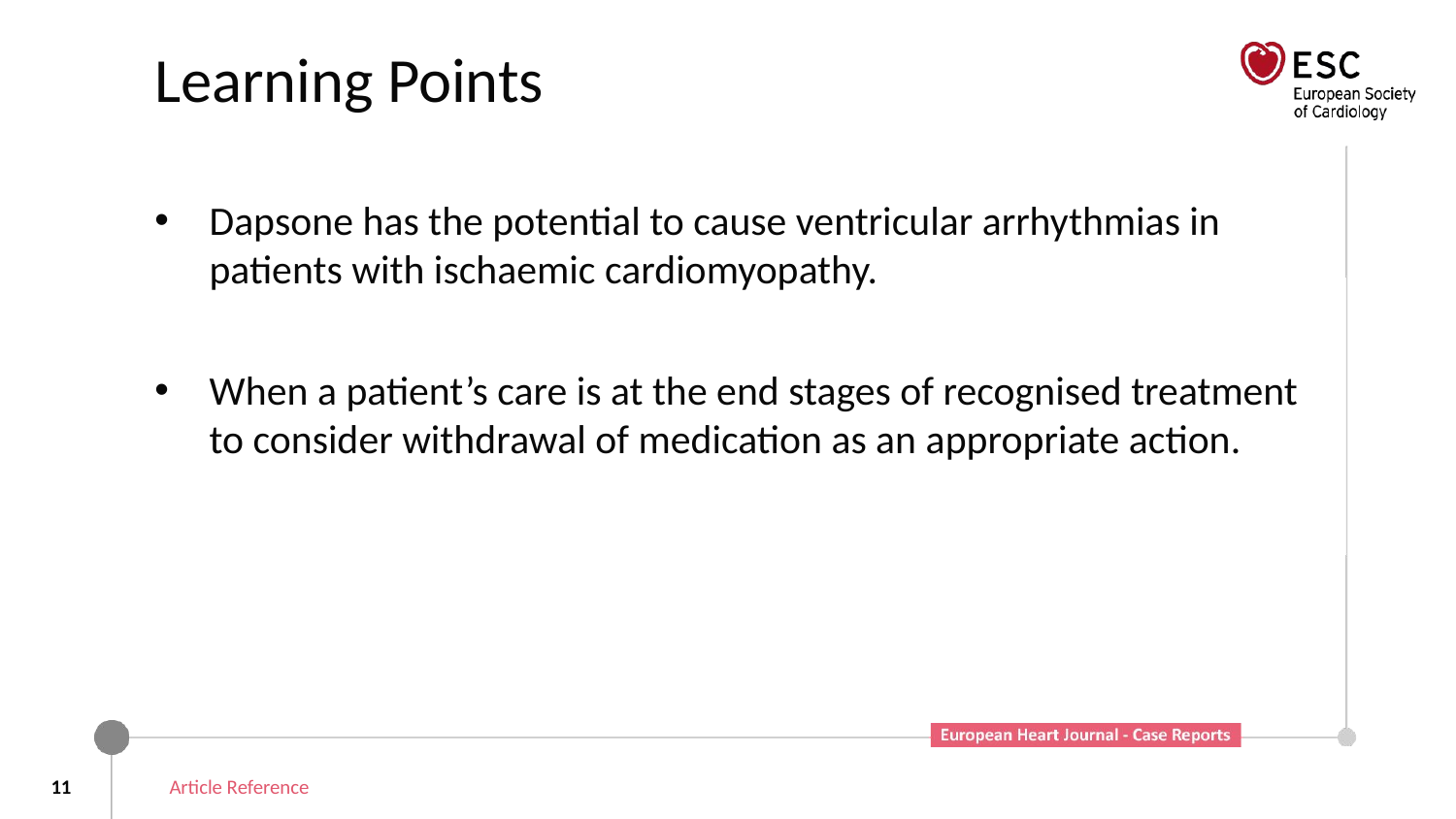

# Learning Points
Dapsone has the potential to cause ventricular arrhythmias in patients with ischaemic cardiomyopathy.
When a patient’s care is at the end stages of recognised treatment to consider withdrawal of medication as an appropriate action.
11
Article Reference

## Slide 12
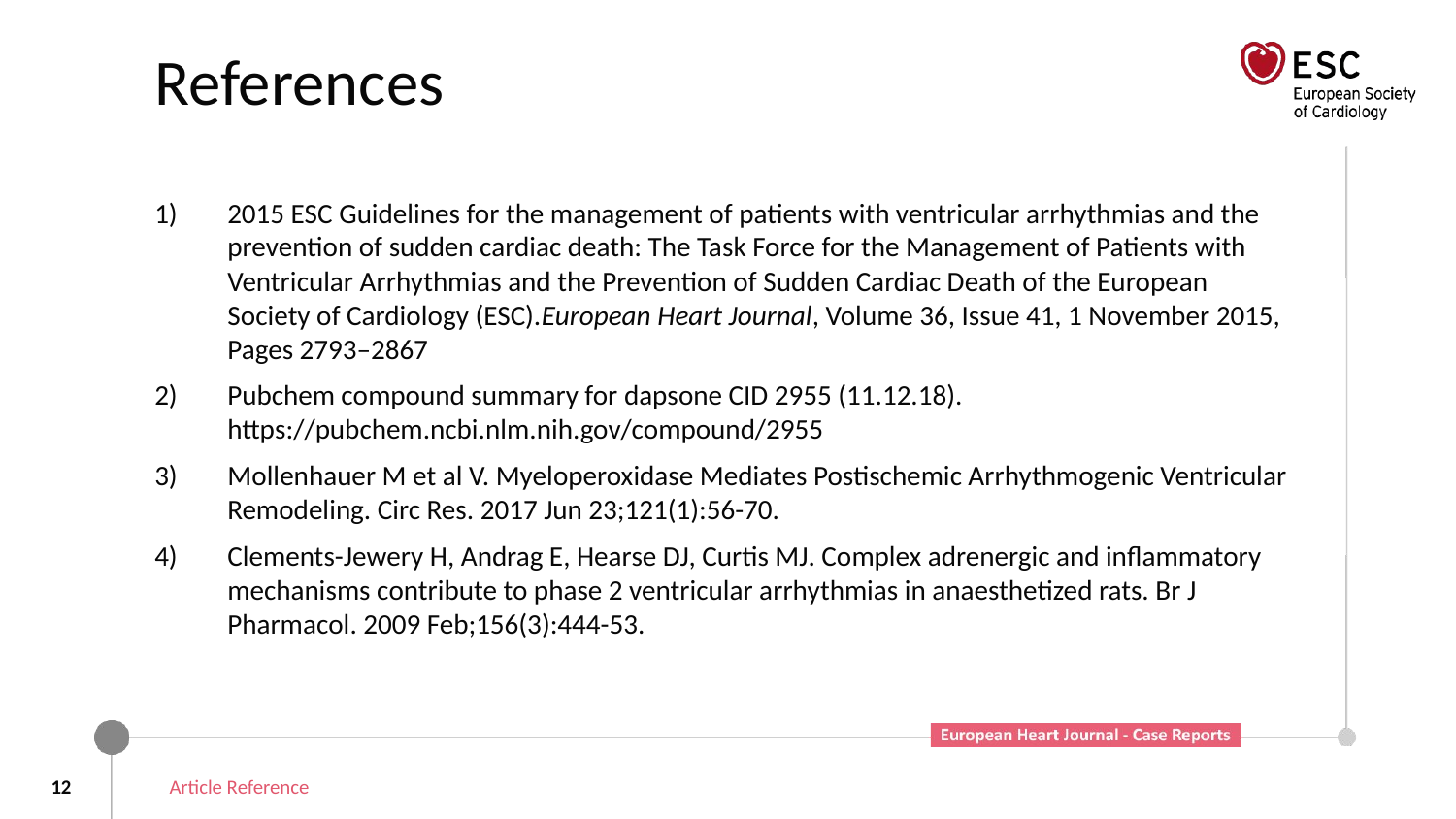

# References
2015 ESC Guidelines for the management of patients with ventricular arrhythmias and the prevention of sudden cardiac death: The Task Force for the Management of Patients with Ventricular Arrhythmias and the Prevention of Sudden Cardiac Death of the European Society of Cardiology (ESC).European Heart Journal, Volume 36, Issue 41, 1 November 2015, Pages 2793–2867
Pubchem compound summary for dapsone CID 2955 (11.12.18). https://pubchem.ncbi.nlm.nih.gov/compound/2955
Mollenhauer M et al V. Myeloperoxidase Mediates Postischemic Arrhythmogenic Ventricular Remodeling. Circ Res. 2017 Jun 23;121(1):56-70.
Clements-Jewery H, Andrag E, Hearse DJ, Curtis MJ. Complex adrenergic and inflammatory mechanisms contribute to phase 2 ventricular arrhythmias in anaesthetized rats. Br J Pharmacol. 2009 Feb;156(3):444-53.
12
Article Reference
